# Integrated weighted gene coexpression network analysis identifies Frizzled 2 (FZD2) as a key gene in invasive malignant pleomorphic adenoma

**DOI:** 10.1186/s12967-021-03204-7

**Published:** 2022-01-05

**Authors:** Zhenyuan Han, Huiping Ren, Jingjing Sun, Lihui Jin, Qin Wang, Chuanbin Guo, Zhen Tian

**Affiliations:** 1 Department of Oral Pathology, Shanghai Ninth People’s Hospital, Shanghai Jiao Tong University School of Medicine, Shanghai, China; 2 National Clinical Research Center for Oral Diseases, Shanghai, China; 3 Department of Oral and Maxillofacial Surgery, Peking University School and Hospital of Stomatology, Beijing, China; 4 Department of Prosthodontics, School and Hospital of Stomatology, Cheeloo College of Medicine, Shandong University & Shandong Provincial Key Laboratory of Oral Tissue Regeneration & Shandong Engineering Laboratory for Dental Materials and Oral Tissue Regeneration, Jinan, Shandong China; 5 Pediatric Heart Center, Xinhua Hospital, School of Medicine, Shanghai Jiao Tong University, Shanghai, China; 6 Clinical Translational Research Center, Shanghai Pulmonary Hospital, School of Life Sciences and Technology, Tongji University, Shanghai, 200092 China

**Keywords:** Invasive malignant pleomorphic adenoma (IMPA), Weighted gene coexpression network analysis (WGCNA), FZD2

## Abstract

**Background:**

Invasive malignant pleomorphic adenoma (IMPA) is a highly malignant neoplasm of the oral salivary glands with a poor prognosis and a considerable risk of recurrence. Many disease-causing genes of IMPA have been identified in recent decades (e.g., P53, PCNA and HMGA2), but many of these genes remain to be explored. Weighted gene coexpression network analysis (WGCNA) is a newly emerged algorithm that can cluster genes and form modules based on similar gene expression patterns. This study constructed a gene coexpression network of IMPA via WGCNA and then carried out multifaceted analysis to identify novel disease-causing genes.

**Methods:**

RNA sequencing (RNA-seq) was performed for 10 pairs of IMPA and normal tissues to acquire the gene expression profiles. Differentially expressed genes (DEGs) were screened out with the cutoff criteria of |log_2_ Fold change (FC)|> 1 and adjusted p value  < 0.05. Then, WGCNA was applied to systematically identify the hidden diagnostic hub genes of IMPA.

**Results:**

In this research, a total of 1970 DEGs were screened out in IMPA tissues, including 1056 upregulated DEGs and 914 downregulated DEGs. Functional enrichment analysis was performed for identified DEGs and revealed an enrichment of tumor-associated GO terms and KEGG pathways. We used WGCNA to identify gene module most relevant with the histological grade of IMPA. The gene FZD2 was then recognized as the hub gene of the selected module with the highest module membership (MM) value and intramodule connectivity in protein–protein interaction (PPI) network. According to immunohistochemistry (IHC) staining, the expression level of FZD2 was higher in low-grade IMPA than in high-grade IMPA.

**Conclusion:**

FZD2 shows an expression dynamic that is negatively correlated with the clinical malignancy of IMPA and it plays a central role in the transcription network of IMPA. Thus, FZD2 serves as a promising histological indicator for the precise prediction of IMPA histological stages.

**Supplementary Information:**

The online version contains supplementary material available at 10.1186/s12967-021-03204-7.

## Background

Invasive malignant pleomorphic adenoma (IMPA) is characterized by high malignancy and invasive growth. It is a subtype of carcinoma ex pleomorphic adenoma (Ca-ex-PA) that arises mainly in the parotid gland, with more than 1.5 mm of cancerous components extending beyond the adenoma capsule into surrounding tissues [1, 2]. Based on the histological morphology of the malignant components, IMPA can be subclassified into the myoepithelial subtype and adenocarcinoma subtype. Briefly, if two or more tumor myoepithelial markers, such as Calponin, S100, or SMA are positively expressed at the same time, IMPA is considered to be a myoepithelial carcinoma subtype. If tumors do not meet the above criteria or are only positive for the epithelial marker Ckpan, they are categorized as adenocarcinoma subtypes [3, 4]. To date, surgical resection combined with radiotherapy and chemotherapy is the main treatment strategy, but the prognosis is poor due to higher local recurrence, distant metastasis and a lower survival rate after surgery [1, 5, 6]. Thus, further research on more accurate molecular targets for early diagnosis and targeted therapy and predictors of a good prognosis of IMPA are urgently needed to develop more efficient therapy that can improve patient survival and quality of life.

A diffusion-weighted imaging (DWI)-based triple-classification radiomics model to characterize intratumoral heterogeneity for preoperative auxiliary diagnosis of pleomorphic adenoma (PA) and a nomogram for predicting the prognosis in an individual with Ca-ex-PA have been established [7]. However, these models are limited to neoplasms and more precise early diagnostic biomarkers are necessary [8]. As bioinformatics analysis is widely applied to cancer research, our previous study first profiled the N^6^-methyladenosine (m^6^A) methylome map in IMPA by methylated RNA immunoprecipitation with high-throughput sequencing (MeRIP-seq) [9]. This study indicated a significant effect of m^6^A modification on IMPA progression, but gene-targeted diagnostic biomarkers are not clear. Weighted gene coexpression network analysis (WGCNA) is a suitable tool to establish free-scale coexpression networks and it is utilized to determine the hub genes based on the correlation between gene modules and clinical characteristics [10, 11]. It has been successfully used in a variety of tumor studies. With WGCNA, potential biomarkers and molecular mechanisms of breast cancer, papillary thyroid carcinoma (PTC) and medulloblastoma (MB) etc. have been identified [12–14], but a gene coexpression network for identifying hub genes closely related to IMPA is still poorly characterized.

Therefore, we evaluated the relationship between the hub gene and tumor grade using WGCNA since histological grade has a strong effect on diagnosis, treatment and prognosis. To the best of our knowledge, our research is the first to utilize WGCNA for the underlying mechanism profiling and biomarker verification of IMPA, which might provide new ideas for increasing diagnosis accuracy and designing efficient strategies for IMPA.

## Methods

### Tissue samples

Ten pairs of IMPA and adjacent normal control tissues for RNA sequencing (RNA-seq) were obtained from Shanghai Ninth People’s Hospital. In addition, another 45 Formalin-fixed and paraffin-embedded IMPA tissues with clinicopathological information were retrieved for verification of hub genes via Immunohistochemistry (IHC). The clinicopathological information of these 45 tissue samples is summarized in Table 1. This study was approved by the ethics committee of Shanghai Ninth People’s Hospital.

**Table 1:** Clinicopathological characteristics of IMPA samples used for FZD2 IHC staining

Characteristics	Category	Number of cases (%)
Age (years)	< 60	14 (31.1%)
	≥ 60	31 (68.9%)
Gender	Female	10 (22.2%)
	Male	35 (77.8%)
Site	Major salivary gland	40 (88.9%)
	Minor salivary gland	5 (11.1%)
Subtype	Adenocarcinoma subtype	21 (46.7%)
	Myoepithelial subtype	24 (53.3%)
Grade	High	29 (64.4%)
	Low	16 (35.6%)
Lymph node metastasis	Yes	18 (40.0%)
	No	27 (60.0%)
Perineural invasion	Yes	28 (62.2%)
	No	17 (37.8%)
Expression of FZD2	Low expression	24 (53.3%)
	High expression	21 (46.7%)

### Histological analyses

All IMPA tissues and adjacent normal controls were fixed in 4% PFA for  > 4 h at 4 °C. Then, these fixed tissues were dehydrated with graded ethanol (70–100%) and embedded in paraffin. Sections (4 μm) were cut on a Leica HistoCore BIOCUT RM2235. Hematoxylin–eosin (H&E) staining was performed following standard procedures. Stained sections were imaged using a Leica LF200 microscope.

### IHC staining

IHC staining was carried out as previously described [15]. In brief, tissue Sections (4 μm) were dewaxed in xylene twice for 2 min each and then rehydrated in a graded series of ethanol (100–70%). Antigen retrieval was performed by boiling sections for 15 min in sodium citrate buffer (10 mM citrate acid, 10 mM sodium citrate, pH 6.0). Then, 5% normal donkey serum was used to block nonspecific antigens. IHC was performed using the Dako EnvisionTM method for antibody incubation and then developed by using the DAB peroxidase substrate kit (Beyotime, P0202). IHC-stained sections were imaged by a Leica LF200 microscope.

Antibodies for IHC included anti-S100 (Dako Z0311, 1:200), anti-Calponin (Dako M3556, 1:100), anti-SMA (Dako M0851, 1:100), anti-CK7 (Dako M7018, 1:50) and anti-CK19 (Dako M0888, 1:100), anti-FZD2 (Bioworld BS3163, 1:50), which were purchased from commercial sources.

### RNA-seq and differentially expressed gene (DEG) identification

Ten pairs of IMPA tissue samples and the surrounding normal control were immediately placed into RNase-free centrifuge tubes and snap-frozen in liquid nitrogen for RNA-seq. Briefly, total RNA was extracted and controlled for quality using a BioAnalyzer 2100 system (Agilent Technologies, Inc., USA). Then, RNA-seq was performed by Illumina NovaSeq™ 6000. EdgeR was performed to normalize the raw data [16] and the DEGs were identified via adjusted p value and Fold change (FC).

### Functional and pathway enrichment analysis

The acquired DEGs were analyzed by the database for annotation, visualization and integrated discovery (DAVID) database (https://david.ncifcrf.gov/) [17, 18] online tool for gene ontology (GO) [19] enrichment analysis and Kyoto Encyclopedia of Genes and Genomes (KEGG) [20] pathway enrichment analysis to identify potential biological functions of DEGs in IMPA.

### Weighted gene co‑expression network construction

The R package ‘WGCNA’ was used to construct a gene coexpression network of DEGs [10]. The correlation strength between nodes was calculated using an adjacency matrix and the formula was as follows:

$${\text{s}}ij = \left| {{\text{cor}}\left( {{\text{x}}i,{\text{ x}}j} \right)} \right|{\text{a}}ij = {\text{S}}ij\beta$$

Briefly, *i* and *j* in the formula were two distinct genes and x*i* and x*j* represented their expression values. s*ij* is Pearson’s correlation coefficient and a*ij* is the strength of the correlation between two genes. The soft-threshold β was 10 in this study. Then, we transformed the adjacency matrix into the topological overlap matrix and performed hierarchical clustering to identify modules with min. Module Size  = 30. Notably, genes without characteristics were assigned to the grey module. Subsequently, gene significance (GS), module membership (MM), module eigengene (ME) and other parameters were calculated to identify the module most relevant with histological grade of IMPA.

### Identification and validation of hub genes

Genes in the selected clinically relevant module were uploaded to the Search Tool for the Retrieval of Interacting Genes/Proteins (STRING) (https://string-db.org/) [21, 22]to analyze the protein–protein interaction (PPI) network of DEGs. The PPI network was visualized via Cytoscape software [23]. KEGG pathway enrichment of these genes was analyzed with the R package ‘ClusterProfiler’ to investigate the biological functions of the module [24]. Of note, the gene with MM  > 0.8 and the highest degree of connectivity was determined to be the hub gene. IHC was further performed to examine the expression level of the hub gene in different clinical stages of IMPA.

### Statistical analysis

Data were expressed as mean  ±  standard deviation (SD) and analyzed using SPSS version 23 statistical analysis package (SPSS Inc., Chicago, IL, USA). Unpaired/paired Student’s t test (two-tailed) was applied to analyze differences between two groups. Pearson’s chi-square test was used to assess differences between the low-grade and high-grade groups. The Pearson’s correlation coefficient analysis was performed to examine the correlation. Statistical significance was described as follows: *p value  < 0.05; **p value  < 0.01; ***p value  < 0.001.

## Results

### Pathologic subtypes of IMPA

**Fig. 1:**
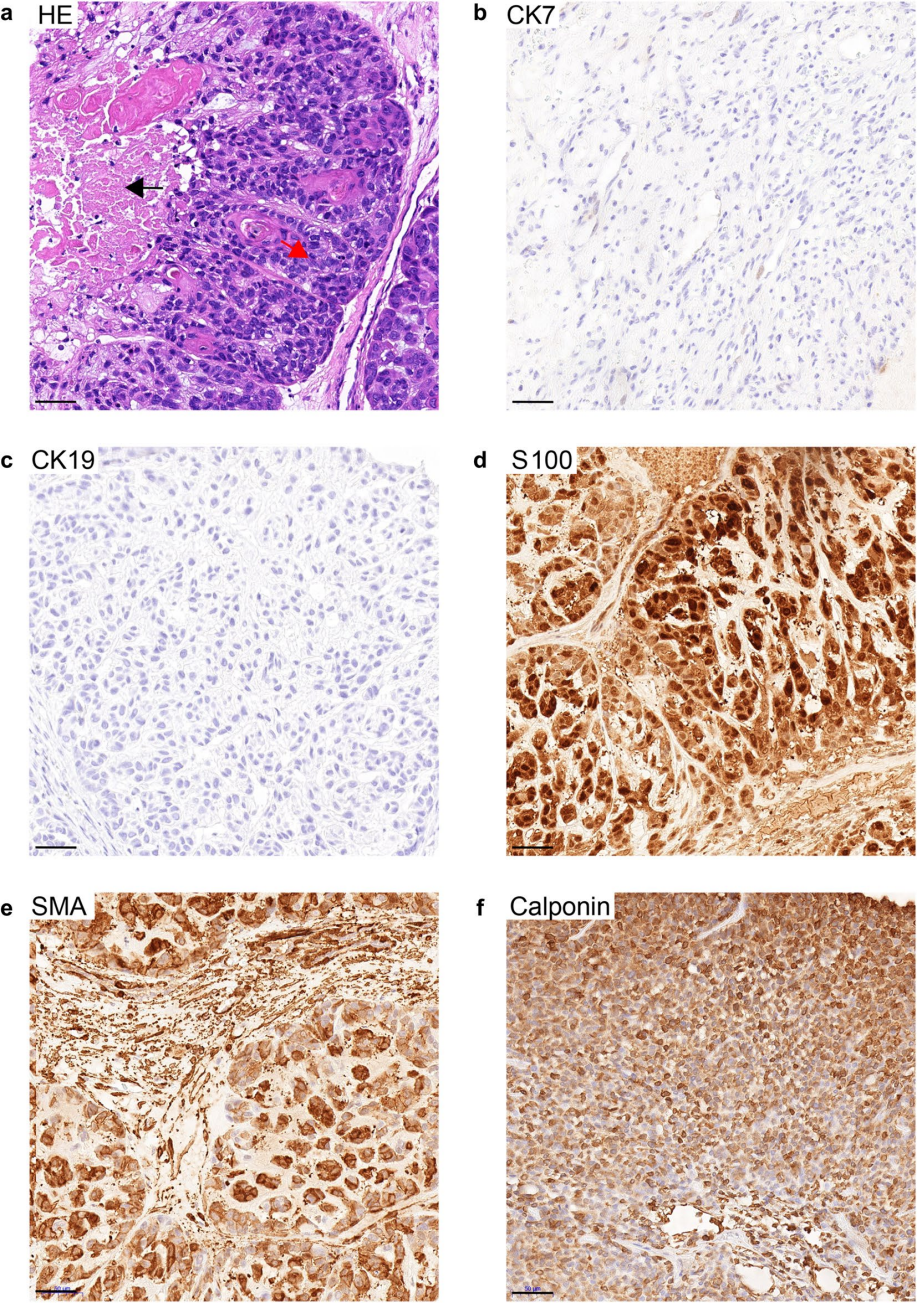
H&E and IHC staining in the myoepithelial subtype of IMPA. **a** H&E staining. Tumor cells were arranged in nodule patterns (red arrow) and separated by thick fibrous stroma. Necrosis (black arrow) was observed in the center of the tumor. IHC staining of CK7 (**b**), CK19 (**c**), S100 (**d**), SMA (**e**) and Calponin (**f**). The myoepithelial subtype of IMPA was positive for S100, SMA and Calponin, but was negative for CK7 and CK19. Scale bar  = 50 μm

**Fig. 2:**
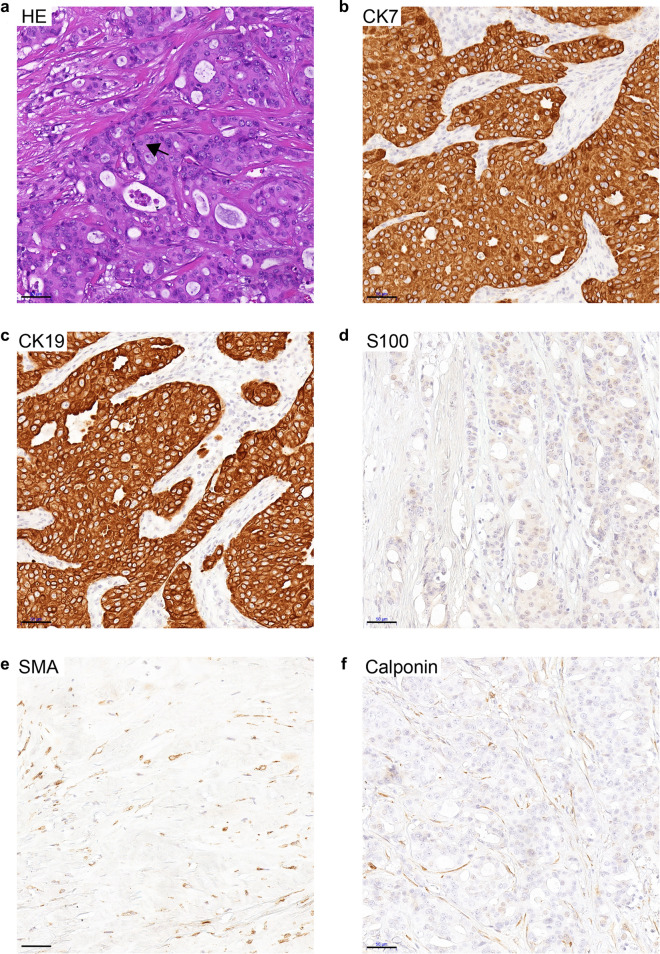
H&E and IHC staining in the adenocarcinoma subtype of IMPA. **a** H&E staining. Tumor cells were arranged in ductal and nested structures (black arrow), with abundant eosinophilic cytoplasm and prominent atypia of cells and nuclei. IHC staining of CK7 (**b**), CK19 (**c**), S100 (**d**), SMA (**e**) and Calponin (**f**). The adenocarcinoma subtype of IMPA was positive for CK7 and CK19, but was negative for S100, SMA and Calponin. Scale bar  = 50 μm

IMPA can be divided into the myoepithelial subtype and the adenocarcinoma subtype [4]. In the 45 IMPA samples for IHC staining, there were 24 myoepithelial IMPAs, which were S100^+^SMA^+^Calponin^+^CK7^−^CK19^−^ (Fig. 1) and 21 adenocarcinoma IMPAs, which were CK7^+^CK19^+^S100^−^SMA^−^Calponin^−^ (Fig. 2).

### Identification of DEGs

**Fig. 3:**
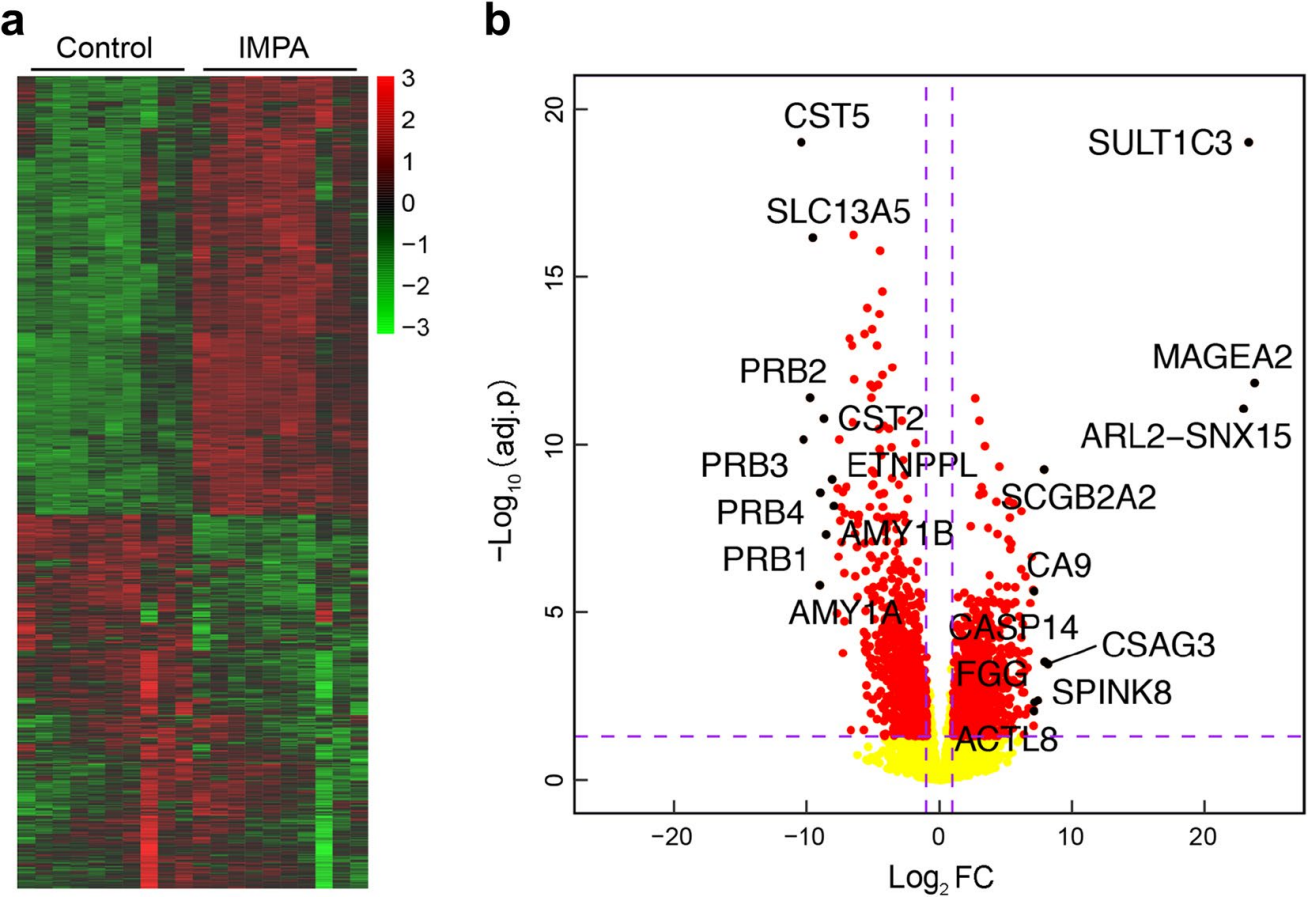
Heatmap and volcano plot displaying the 1970 DEGs in IMPA and adjacent normal controls. **a** The heatmap plot (red, upregulated; green, downregulated). The original data were normalized via the z-score to indexes between − 3 and 3. **b** The volcano plot (adjusted p value  < 0.05, |log_2_FC|> 1; red squares). The p value was adjusted using the Benjamini–Hochberg procedure

Based on |log_2_ FC|> 1 and adjusted p value  < 0.05, a total of 1970 DEGs between IMPA and normal samples were screened out, including 1056 upregulated and 914 downregulated genes (Fig. 3a) [25]. In addition, volcano plots listed the top 10 upregulated and downregulated genes (Fig. 3b, Table 2). More details can be found in Additional file 1: Table S1.

**Table 2:** The top 10 upregulated DEGs and downregulated DEGs

Gene_symbol	Log_2_ FC	adjusted p value
MAGEA2	23.71767826	1.48E−12
SULT1C3	23.27972921	9.83E−20
ARL2-SNX15	22.89375721	8.54E−12
CSAG3	8.151055524	0.000359128
CASP14	7.905691636	0.000307196
SCGB2A2	7.86520853	5.69E−10
SPINK8	7.381732165	0.004260212
FGG	7.100609633	0.004865952
CA9	7.072063302	2.36E−06
ACTL8	7.062711902	0.009017509
AMY1B	− 8.017880777	6.76E−09
ETNPPL	− 8.146282116	1.11E−09
PRB1	− 8.596065483	4.87E−08
CST2	− 8.759797112	1.73E−11
PRB4	− 9.022705183	2.75E−09
AMY1A	− 9.068037572	1.57E−06
SLC13A5	− 9.586123392	6.87E−17
PRB2	− 9.787907831	4.01E−12
PRB3	− 10.29818453	7.14E−11
CST5	− 10.46657151	9.83E−20

### Functional and pathway enrichment analysis of DEGs

**Fig. 4:**
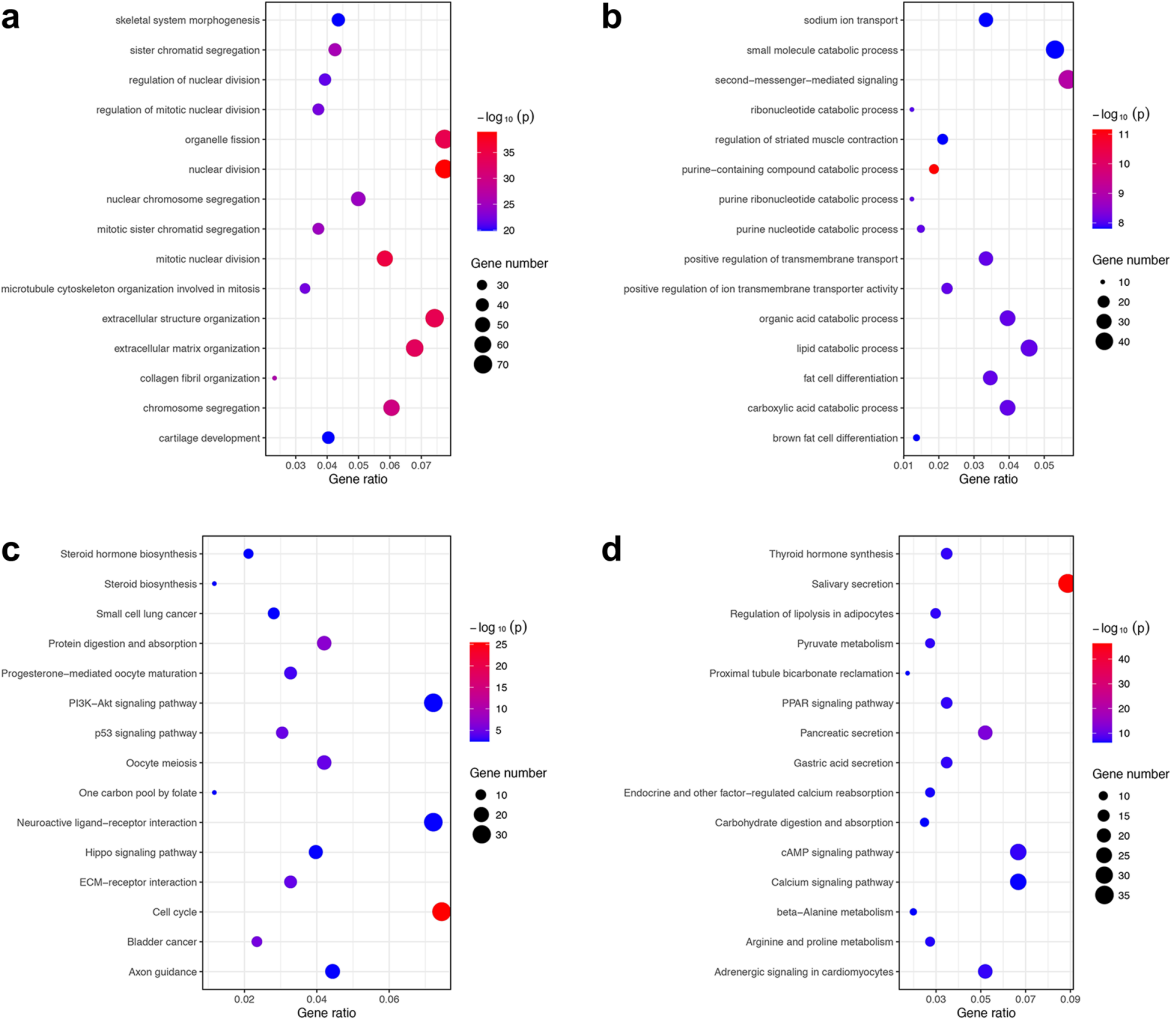
The top 15 ranked GO terms and KEGG pathways according to ‘− log_10_(p value)’. Bubble plots displaying the top 15 enriched GO terms of biological process in 1056 upregulated DEGs (**a**) and 914 downregulated DEGs (**b**). Bubble plots displaying the top 15 enriched KEGG pathways in 1056 upregulated DEGs (**c**) and 914 downregulated DEGs (**d**)

GO and KEGG pathway enrichment analyses were used to verify the potential functional and molecular pathways associated with DEGs. The upregulated DEGs were significantly enriched in biological process of GO terms including nuclear division, organelle fission, extracellular matrix (ECM) organization and chromosome segregation (Fig. 4a). On the other hand, downregulated DEGs were mainly involved in second messenger-mediated signaling, lipid catabolic processes and purine-containing compound catabolic processes (Fig. 4b). In addition, KEGG pathway analysis revealed that upregulated DEGs exhibited enrichments in the cell cycle, ECM-receptor interaction and p53 signaling pathway (Fig. 4c), whereas downregulated DEGs were mainly enriched in salivary secretion, calcium signaling and cAMP signaling pathways (Fig. 4d).

In general, GO and KEGG pathway analyses both suggestd that upregulated DEGs in IMPA exerted functions of promoting tumorigenesis. They were involved in cell cycle progression, ECM organization and tumor-associated signaling regulation. Similarly, downregulated DEGs were revealed to participate in the modulation of second messenger-mediated signaling, which is extremely important for oncogenesis. Notably, the KEGG pathway analysis of downregulated DEGs also showed enrichments in second messenger-mediated signaling (e.g., cAMP and calcium), indicating a critical role of second messengers in IMPA pathogenesis.

### Identification of gene module relevant with IMPA histological grade

**Fig. 5:**
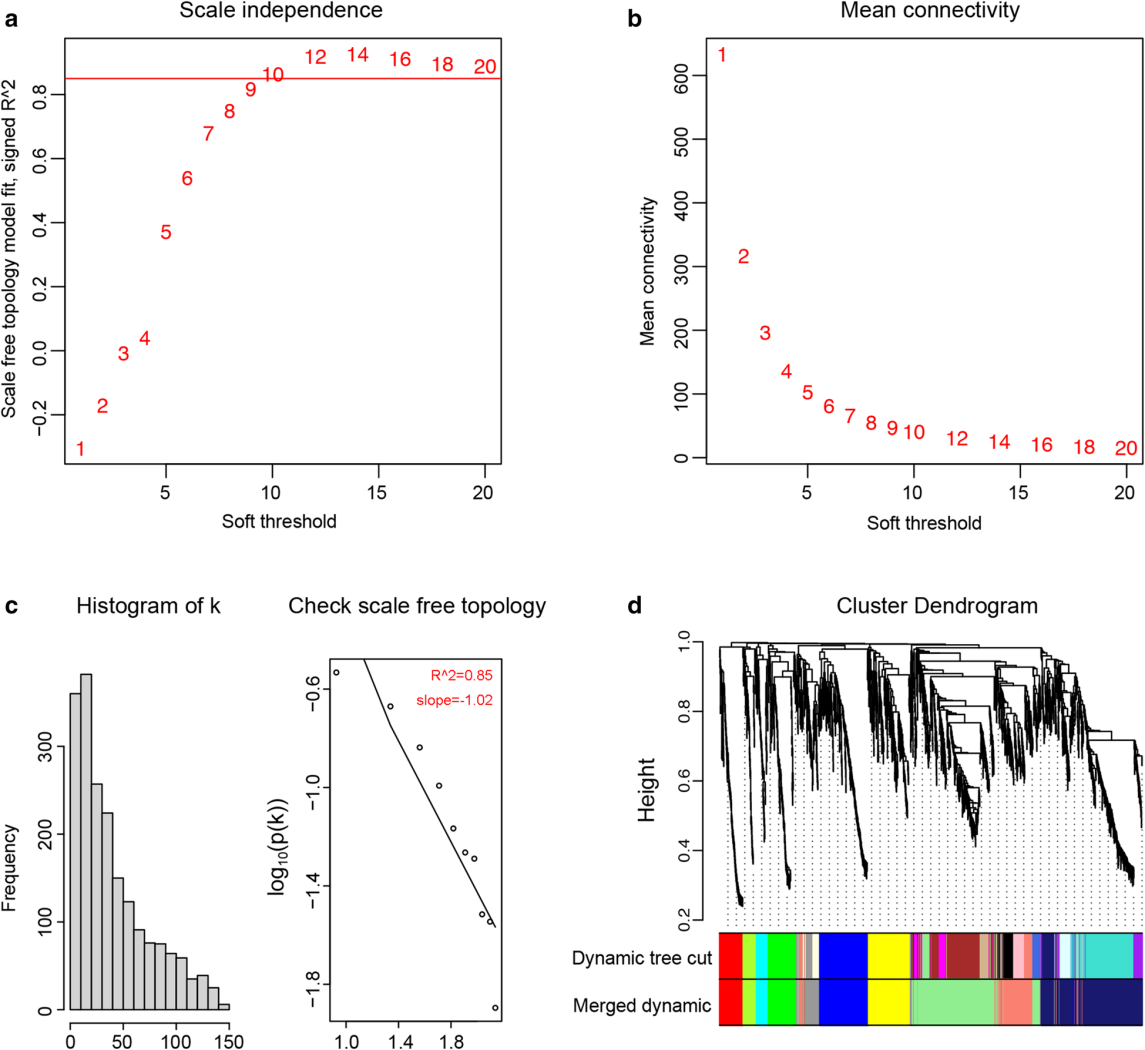
WGCNA network and module detection. **a** Scale-free fit index (y-axis) as a function of soft-thresholding power (x-axis). Power 10 was chosen because the fit index curve flattened out upon reaching a high value (> 0.85). **b** The mean connectivity (y-axis) as a function of soft-thresholding powers (x-axis). **c** Checking the scale-free topology for the soft-thresholding power chosen. **d** Module assignment and cluster dendrogram. Highly interconnected groups of genes were clustered, and modules were represented by distinct colors in the horizontal bar. Eleven modules were identified with the hierarchical clustering tree analysis

**Fig. 6:**
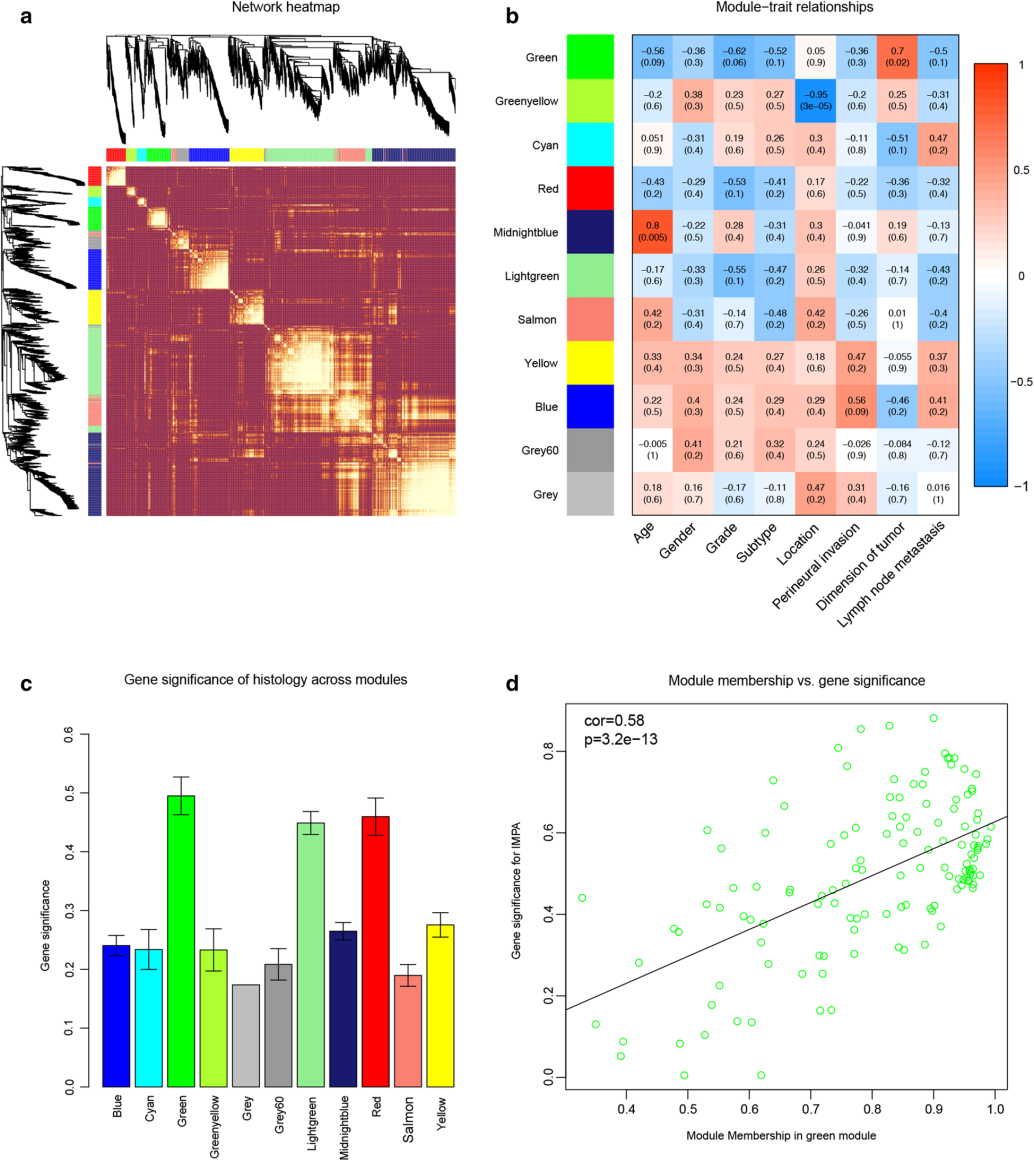
WGCNA of histological grade-related module.** a** Topological overlap matrix plot of the 1970 gene network. Genes in the rows and columns are sorted by the clustering tree in lighter color and show higher overlap. **b** Module—trait relationships between module genes and clinical features of IMPA. **c** GS in modules related to tumor grades. The green module has the highest GS (p  = 1.5e−107). **d** A scatterplot of MM vs. GS module membership (MM) in the green module. There is a strong positive association between MM and GS in this module

A cluster dendrogram of 1970 DEGs was produced via WGCNA based on the criteria of soft-threshold β  = 10 and scale-free R^2^  = 0.85 (Fig. 5a–c). Then, 11 gene modules were identified in the hierarchical clustering, based on a merge cut height of 0.25 and a minimum module size of 30 (Figs. 5d,  6a). The enriched GO terms and KEGG pathways of these 11 gene modules are summarized in Additional file 2: Table S2. It was intriguing to find that there were 6 modules exhibiting enrichment of tumor-associated signaling pathways or GO terms, namely, the cyan module, green module, grey60 module, midnight blue module, salmon module and yellow module. Reasonably, those modules were significantly relevant to the onset and progression of IMPA. The remaining 5 gene modules did not show tumor-related pathway or GO enrichments and thus were not deeply explored in this study.

Among the 6 tumor-associated modules, the green one showed the strongest correlation with the histological grade of IMPA (R  = − 0.62; p  = 0.06; Fig. 6b). Besides, the green module had the highest correlation with histological grade in GS analysis (Fig. 6c). It was noteworthy that MM and GS of the green module were positively related (Fig. 6d). Therefore, the green module was selected for further analysis.

### FZD2 as a promising biomarker of IMPA malignancy

**Fig. 7:**
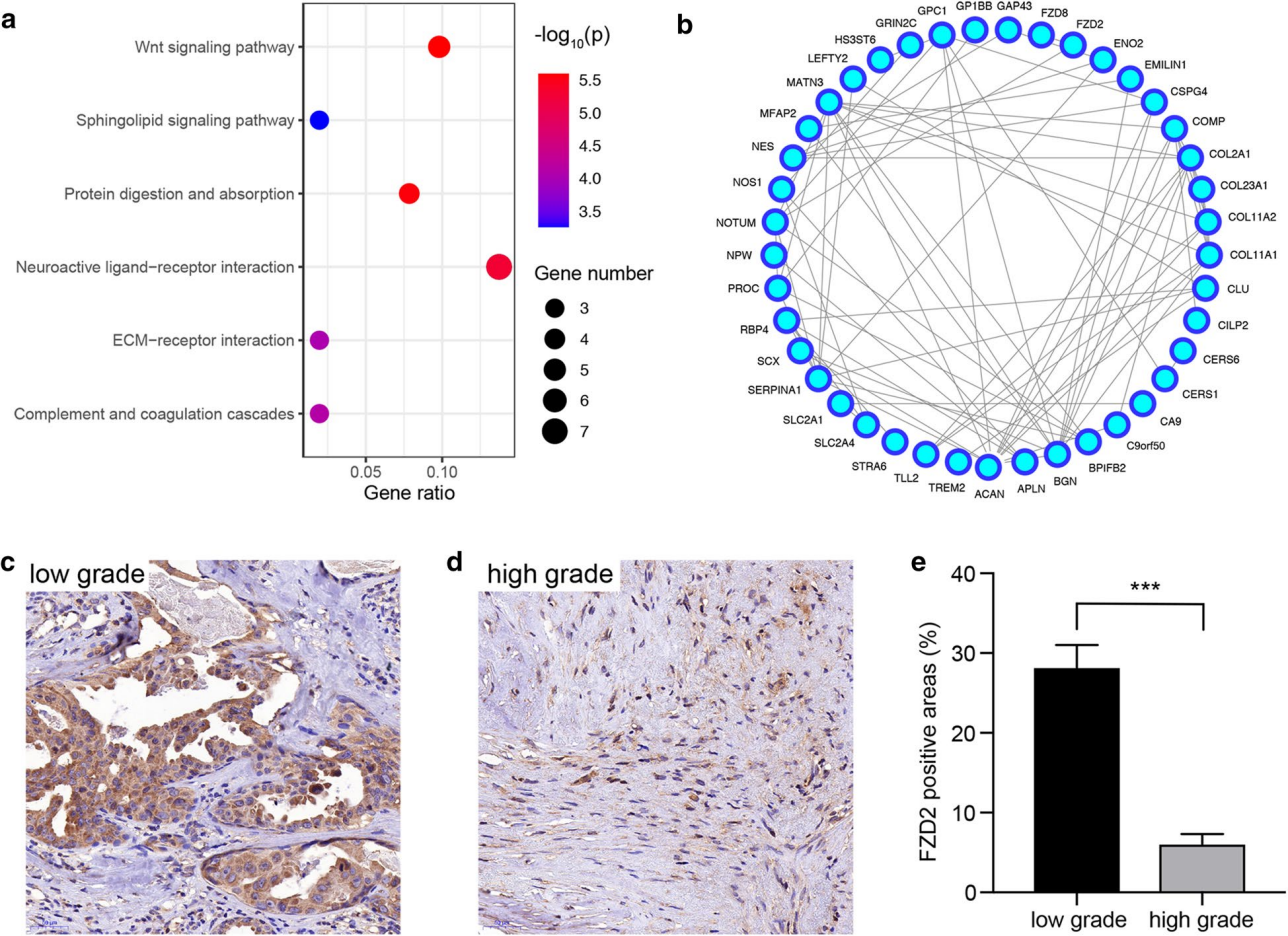
Identification of FZD2 as the hub gene. **a** The bubble plot displaying enriched KEGG pathways in the green module (p value  < 0.05, gene number  ≥ 3). **b** The PPI network of the green module. FZD2 protein staining was higher in low-grade IMPA (**c**) and lower in high-grade IMPA (**d**). **e** Quantification of FZD2 positive areas in low-grade and high-grade IMPAs

KEGG pathway analysis was performed to determine the possible functions of the 132 genes in the green module. As a result, the 132 genes in the green module were primarily enriched in pathways of the Wnt signaling pathway, protein digestion and absorption and ECM-receptor interaction (Fig. 7a). Subsequently, the PPI network of the green module was constructed via the STRING database (Fig. 7b). Notably, FZD2 had the highest MM value and intramodule connectivity among genes involved in the PPI network (Table 3; Additional file 3: S3) and was therefore selected as the core gene. IHC staining of FZD2 revealed that the expression level of FZD2 was negatively correlated with the clinical malignancy of IMPA; that is, the FZD2 protein level was lower in high-grade IMPA than in low-grade IMPA. FZD2 IHC staining was then performed in distinct grades of IMPA tissues and showed identical results (Fig. 7c–e; Table 4). These results suggest that FZD2 might be an ideal indicator for predicting the histological stages of IMPA.

**Table 3:** The intramodule connectivity values of 41 genes in the PPI network

Gene_symbol	Intramodule	Gene_symbol	Intramodule
ACAN	52.61026603	GP1BB	32.09642238
APLN	49.50281239	GPC1	17.64166846
BGN	12.90972979	GRIN2C	42.02411118
BPIFB2	7.554629672	HS3ST6	33.2011835
C9orf50	54.8184171	LEFTY2	20.31343892
CA9	30.21814133	MATN3	48.9472058
CERS1	53.16205839	MFAP2	34.5194173
CERS6	10.1562834	NES	11.29861431
CILP2	52.35240828	NOS1	37.27240571
CLU	44.52886375	NOTUM	52.54787108
COL11A1	17.74844867	NPW	51.46013624
COL11A2	49.91357347	PROC	17.31603023
COL23A1	16.6497107	RBP4	54.28793703
COL2A1	51.2326001	SCX	20.11143562
COMP	52.0206045	SERPINA1	10.12214108
CSPG4	15.87510143	SLC2A1	6.839166971
EMILIN1	51.4866436	SLC2A4	33.68282253
ENO2	33.90715954	STRA6	23.69469976
FZD2	56.74186498	TLL2	19.18490841
FZD8	24.15239668	TREM2	10.95125665
GAP43	15.30427057

**Table 4:** Association between FZD2 expression level and histological grade of IMPA

Group	High expression of FZD2	Low expression of FZD2	χ^2^	p value
High grade	10	19	4.865	0.027*
Low grade	11	5		

Pearson’s Chi-square test

*p value  < 0.05

## Discussion

IMPA is a seriously malignant salivary gland neoplasm with a high risk of recurrence and poor prognosis [1, 2], especially in high-grade tumors [8]. Thus, better biomarkers are needed and the underlying pathogenesis must be clarified for precise diagnosis, targeted treatment and the prognosis prediction of IMPA. Therefore, 10 pairs of IMPA tissue and tumor-surrounding normal tissue samples were subjected to RNA-seq analysis in this study.

In our study, 1970 DEGs were identified according to the criteria of |log_2_ FC|> 1 and adjusted p value  < 0.05. These DEGs included 1056 upregulated genes and 914 downregulated genes. According to the GO analysis results, upregulated DEGs were mainly enriched in nuclear division and extracellular matrix (ECM) organization. The upregulated DEGs, as revealed by KEGG enrichment analysis, participated in pathways such as ECM-receptor interaction and p53 signaling. The ECM provides biochemical and essential structural support for cellular constituents of the tissue and is responsible for cell proliferation, cell adhesion and cell–cell communication [25–27]. Aberrant ECM organization has been reported to be associated with the progression of multiple tumors (e.g., breast cancer and hepatocellular carcinoma) and may likewise contribute to IMPA development [28, 29]. Furthermore, the downregulated DEGs exhibited an enrichment in the GO category of second messenger-mediated signaling and were involved in salivary secretion and calcium signaling pathways. Notably, intracellular calcium ions (Ca^2+^) are well-studied second messengers and they play direct and robust roles in many biological processes. Calcium signaling changes reflect the ‘in or out’ of intracellular Ca^2+^ [30, 31]. Studies have revealed that calcium signaling regulates cancer progression mainly by modulating immune-associated pathways and remodeling the tumor microenvironment [32, 33]. Therefore, there can be dysregulation of intracellular Ca^2+^ and calcium signaling in IMPA.

To determine the hub genes of the 1970 DEGs, WGCNA was performed to construct a gene coexpression network associated with the clinical features of IMPA. Among the 11 identified gene modules, the green module, comprised of 132 DEGs, had the strongest correlation with IMPA stage and grade and was selected for further analysis. Enrichment analysis showed that genes in the green module were enriched in the Wnt signaling pathway, protein digestion and absorption, ECM—receptor interaction and neuroactive ligand receptor interaction. The Wnt signaling pathway is a critical pathway in multiple biological processes [34]. Aberrant activity of the Wnt pathway plays an important role in carcinogenesis, cancer proliferation, metastasis and invasion [35]. Moreover, the activation of protein anabolism is important to high-grade cancers because proteins are required for cell division during proliferation and against cancer-associated cachexia and malnutrition [36]. Studies have reported that neuroactive ligand receptor interactions are involved in tumor immune infiltration and metastasis [37–39]. Therefore, these pathway analysis results may contribute to the comprehensive illumination of the hidden pathogenesis of IMPA.

FZD2 encodes a transmembrane protein with seven transmembrane domains and an extracellular cysteine-rich domain [40, 41]. It can strongly bind to Wnt proteins, which subsequently activates the expression of downstream genes by the Wnt signaling pathway [40, 41]. A study performing a comprehensive analysis of FZD2 in 33 cancer types indicated that FZD2 was associated with high oncogenicity [42]. It was implicated to participate in cancer cell growth, migration and invasion and dominantly affected treatment and prognosis, suggesting the importance of FZD2 in cancer [42]. In oral neoplasms, FZD2 promotes the oncogenesis of oral squamous cell carcinoma [43, 44]. Interestingly, FZD2 was found to act as an inhibitor in salivary adenoid cystic carcinoma, which might be due to the different microenvironment of these tumors [45]. Nonetheless, the role of FZD2 in malignant pleomorphic adenoma has not been reported till now.

In the present study, we showed that FZD2 in this module has the strongest relationship with the grade characteristics of IMPA. Some reports have demonstrated that the expression of FZD2 varies with the different clinical stages and histological grades in cancers [42, 46]. Here, we first found that FZD2 was more highly upregulated in low-grade IMPA than in high-grade IMPA, indicating that FZD2 serves as an ideal indicator for the precise prediction of IMPA histological stages. In general, our study has uncovered an important role of FZD2 in the pathological progression of IMPA. Hub genes of the other 5 tumor-associated modules have also been screened out (Table 5), which might also play a role in IMPA. Further studies are necessary to figure out detailed functions of FZD2 as well as other 5 hub genes in IMPA.

**Table 5:** The hub genes of 6 tumor-associated gene modules

Module	Hub gene
Cyan^a^	IL6
Green	FZD2
Grey60^a^	IL1B
Midnight blue^a^	AURKA
Salmon^a^	SRC
Yellow^a^	CENPE

^a^Obtained by the Maximal Clique Centrality (MCC) algorithm of CytoHubba plugin

## Conclusions

The expression of FZD2 is negatively correlated with the clinical malignancy of IMPA. Moreover, FZD2 was identified as one of the hub genes in the IMPA transcription network and can play a vital role in regulating IMPA progression. In general, FZD2 serves as a promising indicator for predicting the histological stages of IMPA.

## Supplementary Information

**Additional file 1: Table S1.** The 1970 DEGs in IMPA and adjacent normal controls.

**Additional file 2: Table S2. **Table S2. GO and KEGG pathway analyses of 11 gene modules.

**Additional file 3: Table S3. **WGCNA results of the 1970 DEGs.

## Data Availability

The datasets generated and/or analyzed during the current study are from the DAVID (https://david.ncifcrf.gov/). RNA-seq were uploaded to the GEO database (GSE161879 and GSE179895).

## Abbreviations

IMPA: Invasive malignant pleomorphic adenoma
Ca-ex-PA: Carcinoma ex pleomorphic adenoma
RNA-seq: RNA sequencing
DWI: Diffusion-weighted imaging
m^6^A: N^6^-methyladenosine
MeRIP-seq: Methylated RNA immunoprecipitation with high throughput sequencing
WGCNA: Weighted gene co-expression network analysis
PTC: Papillary thyroid carcinoma
H&E: Hematoxylin and eosin
IHC: Immunohistochemistry
FZD2: Frizzled 2
DEGs: Differentially expressed genes
DAVID: Database for annotation, visualization and integrated discovery
PPI: Protein–protein interaction
GS: Gene significance
MM: Module membership
ME: Module eigengene
STRING: Retrieval of interacting genes/proteins
BP: Biological process
ECM: Extracellular matrix
